# Clinical and radiological diagnosis of gallstone ileus: a mini review

**DOI:** 10.1007/s10140-017-1568-5

**Published:** 2017-11-16

**Authors:** Liisa Chang, Minna Chang, Hanna M. Chang, Aina I. Chang, Fuju Chang

**Affiliations:** 1 Department of General Surgery, St. George’s Hospital NHS Trust, London, UK; 2 Faculty of Medicine, Imperial College London, South Kensington Campus, London, UK; 3 School of Cancer & Pharmaceutical Sciences, King’s College London, London, UK; 4 Department of Cellular Pathology, Guy’s & St Thomas’ Hospitals NHS Foundation Trust, London, UK

**Keywords:** Gallstone, Gallstone coleus, Pneumobilia, Rigler’s triad, Small bowel obstruction

## Abstract

Gallstone ileus is a rare cause of bowel obstruction, which mainly affects the elderly population. The associated mortality is estimated to be up to 30%. The presentation of gallstone ileus is notoriously non-specific, and this often contributes to the delay in diagnosis. The diagnosis of gallstone ileus relies on a radiological approach, and herein we discuss the benefits and drawbacks of the use of different modalities of radiological imaging: plain abdominal films, computed tomography, magnetic resonance imaging, and ultrasound scanning. Based on our case experience and review of the literature, the authors conclude that although an effective first-line tool, plain abdominal films are not adequate for diagnosing gallstone ileus. In fact, the gold standard in an acutely unwell patient is computed tomography.

## Introduction

Gallstone disease affects approximately 15% of the population of the UK, often an asymptomatic disease [1]. Gallstone ileus (otherwise known as biliary ileus) is an uncommon presentation of gallstone disease, complicating about 0.5% of gallstone disease [2]. Gallstone “ileus” itself is a misnomer as the underlying pathology is that of mechanical obstruction of the bowel by a gallstone rather than a paralytic ileus as the name suggests. Gallstone ileus accounts for 1–4% of all causes of mechanical bowel obstruction, or up to 25% of all bowel obstruction in the population > 65 years of age [3]. The average age of presentation is 74 years [4, 5]. These patients are usually elderly, frail, and with multiple comorbidities. Gallstone ileus is more prevalent in women than men (ratio of 1:3–7) [3, 4, 6–8] and those of Caucasian ancestry (accounting for 66.5% of gallstone ileus patients) [9]. These figures reflect the old adage taught at all Medical Schools regarding the prevalence of gallstones: Five F’s for “fat, female, fair, fertile and (over) forty (in age)” [10]. The incidence of gallstone ileus is increasing, and this is likely due to the aging population, and better awareness and diagnosis of the condition.

The mortality of gallstone ileus ranges in the literature from 7 to 30% (average 18%) [4, 8, 9, 11–15]. This high mortality is attributed to unmodifiable factors such as elderly or frail population, multiple comorbidities, particularly cardiovascular, respiratory, and endocrine (diabetes and obesity), as well as late presentation from the onset of symptoms (average 4–8 days) [9, 10] or delayed diagnosis. The literature generally describes a median delay between admission and surgical intervention of 2–37 days (range 1–15 days) [5, 9, 15, 16]. Recurrence of gallstone ileus occurs in 5% of cases, with 85% of recurrence within the first 6 months after the initial surgical intervention [7].

## A confounding case of gallstone ileus

**Fig. 1:**
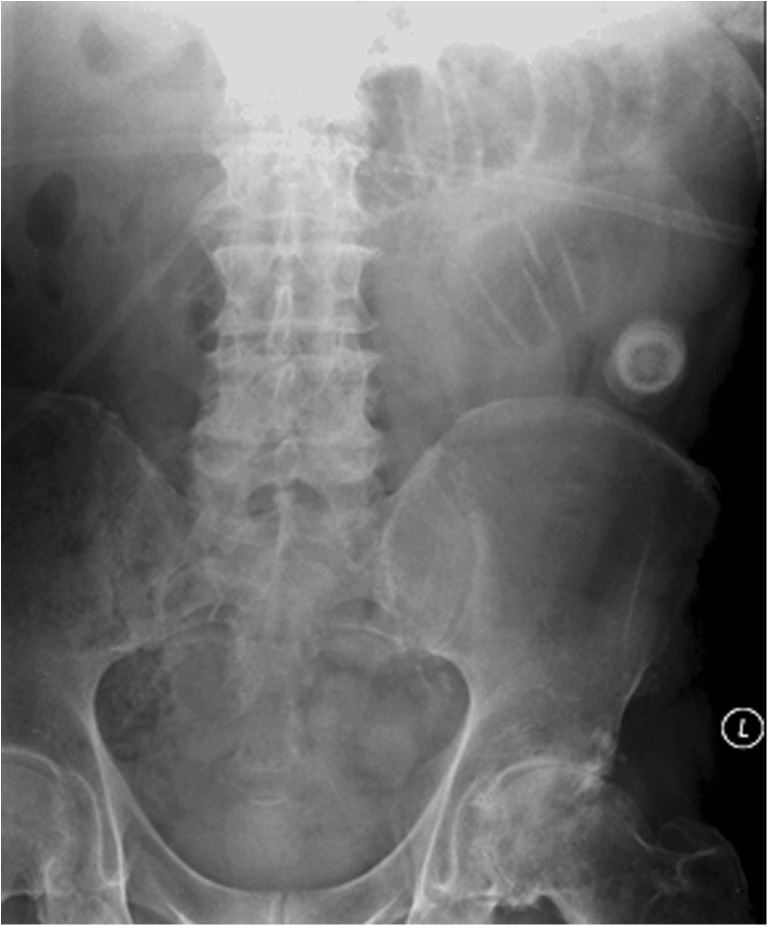
Plain abdominal film at time of admission. Poor quality plain abdominal radiograph obtained in the Emergency Unit, with only part of the abdomen examined. Note the foreign body in the left lower abdomen (ectopic gallstone), with left-sided loops of dilated small bowel. Nil convincing pneumobilia

**Fig. 2:**
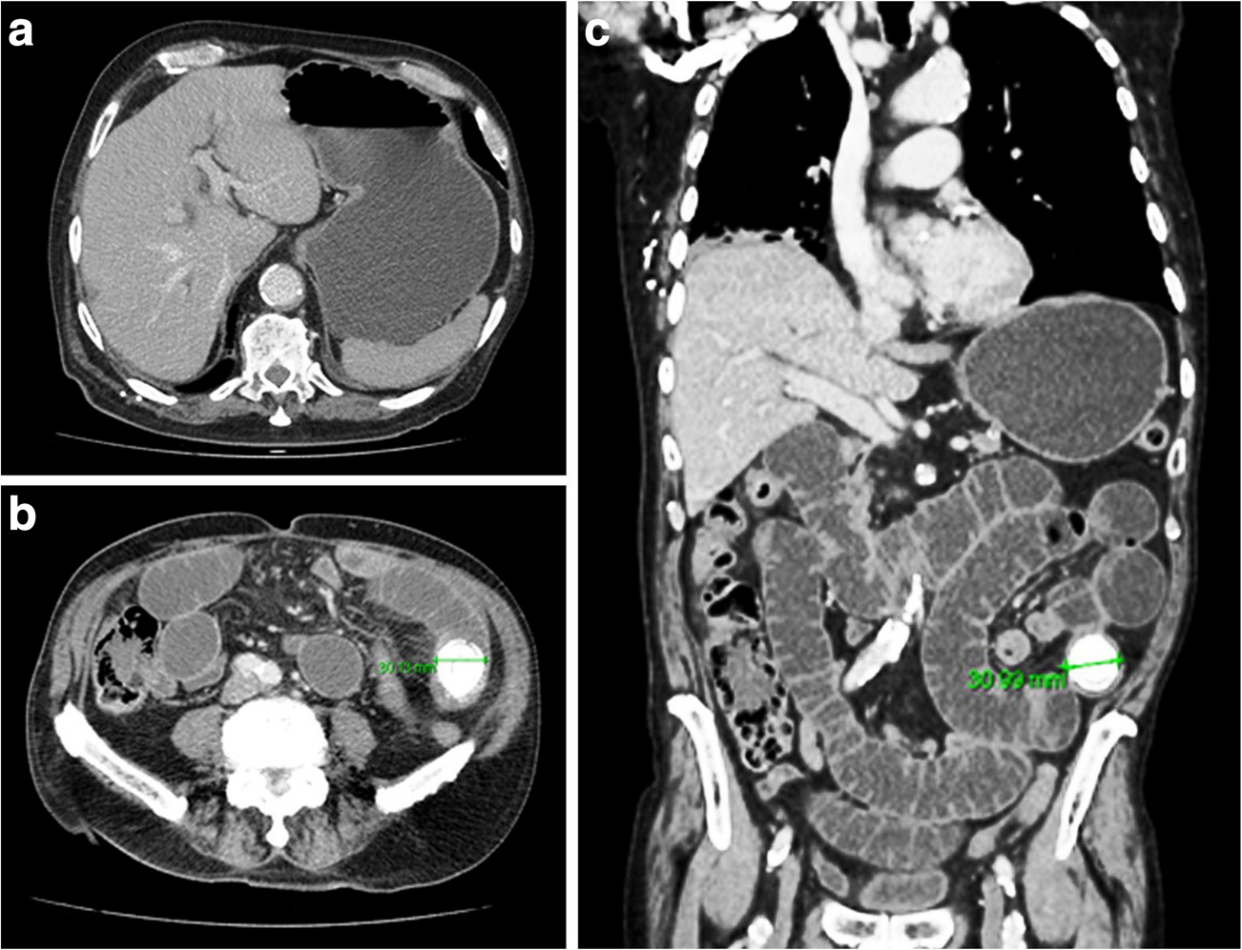
Contrast CT abdomen/pelvis images at time of admission. **a** CT slice demonstrating a dilated, fluid-filled stomach. Nil significant pneumobilia noted. **b**, **c** CT slices demonstrating an obstructing, calcified mid-jejunal intraluminal stone measuring 3 cm in diameter. Evidence of small bowel obstruction noted with dilated jejunal loops above the obstruction. Colon is of normal caliber

Recently, we encountered a case of gallstone ileus. An 82-year-old man was admitted with sudden onset of central abdominal pain and vomiting, describing coffee-ground vomitus. On examination, he appeared distressed with a tender distended abdomen. Blood tests were normal except for chronic microcytic anemia. Patient’s past medical history included the following: chronic obstructive pulmonary disease, ischemic heart disease, gout, stroke, prostate cancer, iron-deficiency anemia, and osteoarthritis. Recent upper gastrointestinal endoscopy demonstrated multiple angiodysplasias in the gastric and duodenal mucosa. The patient was initially managed for an upper gastrointestinal tract bleed (angiodysplasias being a red herring). Erect chest X-ray demonstrated no evidence of perforation, with a poor quality abdominal X-ray (Fig. 1) demonstrating a radio-opaque density in the left iliac fossa with a small loop of distended bowel. These findings were suggestive of gallstone ileus, confirmed by CT scanning (see Fig. 2), and patient underwent laparotomy, enterotomy, and retrieval of gallstone. Patient made an uneventful post-operative recovery and was discharged home.

Due to its uncommon nature, gallstone ileus is often missed or diagnosed late. The lesson from our own case experience has led us to look at the clinical and radiological presentation and diagnosis of gallstone ileus, in order to address issues contributing to the delays in diagnosis.

## Pathophysiology

The definition of gallstone ileus is the obstruction of bowel (whether small intestine or large intestine, also called “gallstone coleus”) due to the impaction of one or more gallstones. To achieve this, the impacted stones are usually greater than 2–2.5 cm in diameter [3, 5, 6]. Smaller stones pass through the lumen of the bowel as “rolling stones” and rarely cause obstruction. However, cases have been reported of multiple stones causing obstruction as an inspissated mass [15, 17].

Common places for gallstones to be lodged include the ileum and ileocecal valve due to the anatomical narrow lumen in 60% of cases, jejunum in up to 16%, stomach in 15%, and colon (gallstone coleus) in 2–8% of cases [18–22]. Rare cases of gallstone impaction have been reported at sites of strictures, e.g., Crohn’s or diverticulitis, and stenosis, e.g., at the neck of a Meckel’s diverticulum [18, 23].

Obstructing gallstones generally migrate to the bowel via bilioenteric fistulas, the most common of which is a fistulous connection from the gallbladder to the duodenum (85% of cases) [3]. These fistulas form following chronic erosion by a stone or recurrent/chronic inflammation of the gallbladder wall, i.e., calculous cholecystitis. However, reports demonstrate that only 25–72% of gallstone ileus cases demonstrate a previous history of gallstone disease [3, 9, 16, 24]. Other types of fistulas include hepatoduodenal, choledochoduodenal, cholecystogastric, cholecystojejunal, and cholecystocolonic [3]. Rare case reports have demonstrated enteric fistulation into bowel secondary to gallbladder malignancy [25].

Other, rarer, mechanisms of enteric gallstone migration include the passage of gallstones through the ampulla of Vater followed by in situ growth, or inspissation of multiple small stones, or the inadvertent iatrogenic migration of gallstones during manipulation of the gallbladder or ducts (e.g., during ERCP or a cholecystectomy) [26].

There are two, rare subtypes of gallstone ileus, which we will discuss herein: gallstone coleus and Bouveret’s syndrome. Gallstone coleus is an extremely rare cause of mechanical large bowel obstruction, with few cases reported in the literature. It may be the result of gallstone erosion into and passage through the small bowel (and thus the ileocecal valve), or by direct erosion into the large bowel. The latter generally involves the transverse colon, which is in close anatomical proximity to the gallbladder [21, 27, 28]. Although gallstone coleus due to transverse colon obstruction has been reported [28], by far the most common reported site of gallstone impaction is the sigmoid colon [23, 29–31], often in association with diverticular disease [32–35]. Stone impaction in gallstone coleus, as with gallstone ileus, may occur due to sheer size of the obstructing stone [27], or secondary to pathological narrowing of the bowel lumen due to diverticular strictures [36, 37], prior pelvic irradiation [38], or previous surgical intervention [39].

Bouveret’s syndrome is an even rarer type of gallstone-associated gastrointestinal obstruction, which occurs when a gallstone lodges in the duodenum, causing gastric outlet obstruction [40–42]. These gallstones are typically very large in order to cause obstruction at an anatomically wide part of the gastrointestinal tract [40–42]. This is estimated to occur in 1–3.5% of gallstone ileus [3, 13, 19].

## Presentation and clinical diagnosis

The clinical presentation of gallstones is notoriously non-specific. The symptoms and signs differ depending on the site of gallstone impaction, and the duration of symptoms can vary significantly from hours to days to weeks, as previously alluded to [7, 15, 43]. Most commonly, gallstone ileus resembles small bowel obstruction of any cause. Symptoms and signs are often intermittent and may include the following:

- Abdominal pain—usually generalized and colicky/cramping due to bowel obstruction [15].
- Nausea and vomiting—often follows abdominal pain. The vomitus may be bilious (small bowel obstruction, SBO), feculent (large bowel obstruction, LBO), or may appear “coffee-ground” (see below).
- Abdominal distension—this is a very variable sign. Clinical examination may elicit tinkling/high-pitched bowel sounds.
- Constipation—this is variable (patient may initially continue to pass stool or flatus, but this can become absolute constipation).
- Signs of upper gastrointestinal bleeding including hematemesis, coffee ground vomitus or melena due to the erosion of gastroduodenal artery [13].
- Signs of bowel perforation ± peritonitis are extremely rare as first presentation of gallstone ileus [23].
- Patients may also relate a history of intermittent right upper quadrant pain, weight loss, anorexia, or early satiety. Acute cholecystitis is present in 10–30% of patients at time of gallstone ileus [15].
- Rare presentations of gallstone ileus reported in the literature include gangrenous appendicitis with a gallstone impacted at the base of the appendix [44], or intussusception with the stone serving as a lead point [45, 46].
- Most episodes of gallstone ileus tend to present as acute/subacute. However, a rare form of chronic gallstone ileus has been described in the literature [47]. Karewsky syndrome is characterized by intermittent episodes of pain due to gallstone(s) lodged in the bowel over a protracted period of time [47].

Blood tests at time of presentation with gallstone ileus tend to be unremarkable (unless there are signs of intra-abdominal sepsis), and the diagnosis is usually made with radiological imaging.

## Diagnostic imaging

### Plain abdominal film

Plain abdominal film radiography, or abdominal X-ray (AXR), is an ideal modality in the emergency setting. It is a quick and technically easy diagnostic test, and the radiation dose is low at 0.7 mSv per film [39]. AXR may be performed upright or supine, with the latter being the conventional method. It is often the first-line investigation used for the diagnosis of gallstone ileus, or indeed any case of bowel obstruction. The sensitivity of AXR for the diagnosis of gallstone ileus is between 40 and 70% [3, 46, 48], and its positive predictive value approaches 80% in patients with high-grade intestinal obstruction. Rigler described his famous triad of radiological signs in 1941 for gallstone ileus on plain film: air within the biliary tree (pneumobilia), signs of small bowel obstruction, and ectopic radio-opaque gallstones [49]. Rigler’s triad is pathognomonic for gallstone ileus, but in most cases, only two signs out of the triad are present. In the literature, only 14–53% of cases present with the full criteria [3, 16, 43, 50, 51].

One of the most prominent signs of gallstone ileus on AXR is that of any other cause of mechanical bowel obstruction. In the case of small bowel obstruction, supine AXR shows multiple loops of dilated small bowel (in an upright AXR, there are multiple air fluid levels within dilated bowel) with paucity of air in the large bowel and/or rectum [48]. In the case of gallstone coleus, the large bowel is dilated, and depending on the competency of the ileocecal valve, the small bowel may be decompressed [48]. In the rare cases of Bouveret’s syndrome of gastric outlet obstruction, AXR may sometimes demonstrate a prominent gastric shadow [52].

Gallstones are notoriously difficult to visualize on plain film radiography, with only 10–20% of stones containing enough calcium to be radio-opaque [8, 53]. Of those stones not visualized, the vast majority are radiolucent (typically associated with cholesterol stones), but a number may be veiled by obesity, or superimposed by bony structures or fluid-filled bowel [17].

Although pneumobilia is a sign highly suggestive of gallstone ileus, it is not isolated to gallstone ileus. Its presence on AXR has been described in sphincter of Oddi dysfunction, as well as post-ERCP. Several studies have also shown a lack of consistency in the identification of pneumobilia. It is often a subtle radiological sign and may be missed by the attending physician, only identified in retrospect to be present in up to 50% of plain AXRs [3, 8, 15, 16, 43, 50, 54, 55].

Since Rigler defined his triad in 1941, two further radiological signs for gallstone ileus have been described: change in the location of a previously noted gallstone (Rigler’s tetrad) [24], and a dual air-fluid level in the right upper quadrant (herein Rigler’s pentad). The latter was described by Balthazar and Schecter in 1978 [23, 24, 55] as either a dual air-fluid level on upright AXR, or a double air bubble on supine AXR, and it is estimated to be present in up to 24% of patients at admission [15].

A further literature search has revealed two signs seen on AXR with oral contrast (either water soluble contrast or barium): Forchet sign, when contrast passes around a radiolucent calculus (forming a snake’s head of contrast with a clear halo of the calculus) [24] and Petren sign (passage of contrast from bowel to gallbladder through a fistulous connection) [3]. In addition to reports of oral contrast for gallstone ileus, there are also reports of the use of contrast enemas for the identification of gallstone coleus [23, 38]. We, as many authors before us, emphasize that oral or rectal contrast should not be routinely used in suspected gallstone ileus. In cases of perforation, barium leak can lead to barium peritonitis (voided by the use of water soluble contrast). In addition, in cases of bowel obstruction, excessive oral fluid intake can aggravate the symptoms including vomiting with a high risk of aspiration of contrast. Furthermore, gallstone ileus nowadays is easily diagnosed on CT scanning, negating the need for oral contrast AXRs.

In summary, there are five commonly seen radiological signs (as defined above) visible on plain AXR, the presence of a combination with is almost diagnostic for the presence of gallstone ileus.

### Computed tomography

Computed tomography (CT) scanning is widely accepted as the investigation of choice in bowel obstruction, or indeed any other cause of acute abdomen. CT scanning in unwell patients is relatively fast and becoming more widely available [3, 14, 19, 24, 43, 56]. The radiation dose involved is 10 mSv [39], roughly 10 times that of an AXR. CT scan of the abdomen provides exquisite anatomical detail not afforded by AXR, including abnormal fistulous connections [7], gallbladder anatomy, and even small stones/gallstone sludge in the gallbladder, biliary tree or ectopic stones elsewhere in the GI tract. The diagnostic signs for gallstone ileus on CT scanning has been defined by Yu et al. in 2005: signs of SBO, ectopic gallstone, abnormal gallbladder, e.g., complete air collection, presence of air-fluid level, or fluid accumulation within an irregular wall [43].

Contrast-enhanced CT is considered the gold standard for the diagnosis of intra-abdominal pathologies; however, non-contrast CT has notable benefits: it conveys no risk of contrast allergy, can be used to investigate patients with renal impairment, and importantly, non-contrast CT identifies ectopic gallstones irrespective of the degree of calcification [8, 23]. Contrast-enhanced CT for gallstone ileus has a sensitivity of 90–93% [3, 14, 19, 24, 43], specificity of 100% [19, 43], and accuracy of 99% [19, 43] in patients presenting with acute small bowel obstruction. In addition, CT scanning has the benefit of being able to define the cause and level of bowel obstruction (guiding operative management) and defining the size and structure of the ectopic stone. Moreover, contrast CT is invaluable in detecting bowel edema, inflammation, and ischemia thus helping to identify potential complications of gallstone ileus, as well as identifying alternative causes for patients’ symptoms [57].

CT contrast may be administered as intravenous (IV) or oral preparations. Oral contrast may be used to better evaluate the anatomy of the bowel, and the presence and degree of obstruction (location of transition zone or the filling defect of an ectopic gallstone) [43]. In the context of gallstone ileus, oral contrast may also visualize fistulous connections between the gallbladder and bowel through contrast accumulation within the gallbladder [58]. However, in an acute abdomen, IV contrast is often preferred, as it enhances the bowel as well as the other abdominal viscera, which may allow exclusion of alternative diagnoses of acute abdomen. As with the use of oral contrast in plain abdominal films, excessive fluid intake in the form of oral contrast in bowel obstruction may aggravate the symptoms, and pose an aspiration risk. Furthermore, the existing intestinal fluid and gas within an obstructed bowel already offer a natural negative contrast [43]. The authors note that the administration of oral contrast in acute bowel obstruction causes a delay in CT imaging as time must be allowed from time of contrast ingestion for the contrast to pass through the bowel [58]. The standard protocol for CT scanning in suspected bowel obstruction is with IV contrast using portal venous phase [8].

Gallstones demonstrated on CT scanning consistently fall into the following categories [59]:

- Dense opacification (calcified gallstones) in 48.3%
- Slight opacification in 11.5%
- Rim opacification in 21.8%
- Radiolucent (generally pure cholesterol stones) in 14.9%
- Composite stones of calcium, cholesterol, and bile pigments may be missed on CT scanning due to isoattenuation relative to bile/fluid. This may occur in up to 25% of cases [15, 59]. A study by Barakos et al. reports several cases of isoattenuating tones relative to the fluid, which has accumulated in obstructed bowel. These stones were missed on CT but were later demonstrated by ultrasound scanning or postoperatively [59].

There are several reports of CT scanning underestimating the size of stones in gallstone ileus [40]. In IV contrast-enhanced CT scanning, this may be due to radiolucency at the periphery of the stones, or stones embedded in the mucosa of the bowel wall [8, 24, 40]. Furthermore, the drawback of IV contrast-enhanced CT scanning in gallstone ileus is the difficulty in defining some radiolucent stones or rim-calcified stones [43, 60]. Intraluminal radiolucent stones may resemble soft-tissue densities (isoattenuation), e.g., a mass [53, 61] or an intussusceptum as reported by Prasad et al. [46]. On the contrary, in contrast-enhanced small bowel, rim-calcified (thus rim-enhancing on CT) stones may go undiagnosed given their strong resemblance to contrast-enhanced small bowel (the radiolucent center of the calculus resembling bowel lumen). Such rim-enhancing stones may be calcified completely (encircling the stone) or as an arc, and occur in approximately 22% of cases of gallstone ileus [59, 60].

In summary, the identification of gallstones on CT scanning is complicated by variability in gallstone composition and structure. Its applicability in this condition is highly dependent on a high index of suspicion, and the skill of the observer. However, overall CT scanning is a powerful tool for the diagnosis of bowel obstruction, and thus one of its rarer causes, gallstone ileus [57].

### Abdominal ultrasonography

Abdominal ultrasonography (US) is the method of choice for the detection of gallstone disease, with efficacy of greater than 95% [1, 53, 62]. It is a low cost, non-invasive tool with a notable benefit of no radiation exposure to the patient. However, it is rarely used for diagnostic purposes in unwell patients with acute abdomens. In cases of bowel obstruction, it is a technically difficult scan due to patient discomfort, and gaseous/fluid distension of bowel. There is evidence that with an experienced sonographer, ultrasonography may be used demonstrate the signs of Rigler’s triad in gallstone ileus including location of a lodged stone in the bowel lumen [13, 15, 24, 51, 63]. It can also demonstrate any residual cholelithiasis or choledocholithiasis. Furthermore, the use of AXR in combination with ultrasonography can increase the sensitivity of the latter to 74% by identifying signs such as pneumobilia [64].

Table 1 shows the radiological signs of gallstone ileus identified by each imaging modality as a percentage [43, 50, 54, 64]. In comparison to AXR and US, CT scanning is evidently better at identifying these signs.

**Table 1:** Radiological signs of gallstone ileus identified by each imaging modality

	AXR	Ultrasound	CT
Bowel obstruction	88.89%	44.44%	96.3%
Pneumobilia	36–37.04%	55.56%	50–88.89%
Ectopic gallstone	33.33–42%	14.81%	81.48–92%
Rigler’s triad	14.81–35%	11.11%	77.78%
Bilioenteric fistula	–	–	11.11%

Data from references 43, 50, 54, 64

### Magnetic resonance imaging

Magnetic resonance imaging (MRI) is the gold-standard modality for visualizing the biliary tree, detecting even small gallstones like microcalculi (< 3 mm diameter) missed on ultrasound scanning [62], or radiolucent and isoattenuating stones missed on conventional AXR or CT scanning [57]. The sensitivity of magnetic cholangiopancreatography (MRCP) in the diagnosis of gallstones is 97.7% [62], and it provides exquisite anatomical detail of the biliary tree. Although MRI scanning can demonstrate Rigler’s triad in almost 100% of cases [57] compared to 77.8% with CT scanning, it does not play a role in the acute setting as it is more time-consuming and less readily available than CT.

A potential use for MRI in gallstone ileus has been investigated. It may be useful in estimating the risk or tendency of chronic calculus gallbladder to fistulate into bowel [57]. MRI scanning is able to demonstrate signs such as chronic calculus cholecystitis, large gallstones (> 2 cm), gallbladder wall thinning or blurring or loss of the fat line between the gallbladder and duodenum. Further evidence for its use is required.

## Management

Management of gallstone ileus is as for most other causes of small or large bowel obstruction. Initial management begins with the “drip and suck” regimen (nasogastric tube for decompression and intravenous fluids for rehydration). There are reports in the literature for spontaneous resolution of gallstone ileus with patients passing stones per rectum [65]. However, in the vast majority of patients, surgical management is generally required [20, 66]. In addition to the clinical assessment of a patient, radiological indications for urgent surgical intervention include established or impending bowel perforation, signs of bowel ischemia, or evidence of significant intra-abdominal bleed (for control of hemorrhage). The different surgical approaches have been discussed elsewhere and will not be discussed further herein. Surgical management in this aforementioned cohort of patients (typically elderly, frail with multiple comorbidities) has a high risk of morbidity and mortality, and efforts are often directed at less invasive management. For example, several attempts have been reported in the literature for the use of endoscopy (namely colonoscopy) with or without lithotripsy in the context of gallstone coleus [29, 31, 34, 67].

## Conclusion

Gallstone ileus is a rare complication of gallstone disease with a variable and non-specific clinical presentation. It requires a high index of suspicion, particularly in elderly patients presenting with signs of small bowel obstruction. The use of radiological imaging is invaluable in the diagnosis of gallstone ileus. These authors recommend a low threshold for investigation. There is evidence for the use of AXR as a quick first-line investigation; however, CT scanning is a powerful and gold-standard tool to diagnose the condition and to guide its management.
